# Quantification of Radiation Injury on Neutropenia and the Link between Absolute Neutrophil Count Time Course and Overall Survival in Nonhuman Primates Treated with G-CSF

**DOI:** 10.1007/s11095-020-02839-3

**Published:** 2020-05-21

**Authors:** John Harrold, Per Olsson Gisleskog, Isabelle Delor, Philippe Jacqmin, Juan Jose Perez-Ruixo, Adimoolam Narayanan, Sameer Doshi, Andrew Chow, Bing-Bing Yang, Murad Melhem

**Affiliations:** 1 Department of Clinical Pharmacology, Modeling and Simulation, Amgen Inc., Thousand Oaks, California USA; 2 SGS Exprimo NV, Mechelen, Belgium; 3 Present Address: POG Pharmacometrics, Hampshire, UK; 4 Present Address: Janssen Research & Development, Valencia, Spain; 5 Present Address: Rigel Pharmaceuticals Inc., South San Francisco, California USA; 6 Present Address: Vertex Pharmaceuticals, Boston, Massachusetts USA; 7 Present Address: Seattle Genetics, Bothell Washington, Massachusetts USA

**Keywords:** Acute radiation syndrome, filgrastim, neutropenia, overall survival, pegfilgrastim

## Abstract

**Purpose:**

To model absolute neutrophil count (ANC) suppression in response to acute radiation (AR) exposure and evaluate ANC time course as a predictor of overall survival (OS) in response to AR exposure with or without treatment with granulocyte colony-stimulating factor in nonhuman primates.

**Methods:**

Source data were obtained from two pivotal studies conducted in rhesus macaques exposed to 750 cGy of whole body irradiation on day 0 that received either placebo, daily filgrastim, or pegfilgrastim (days 1 and 8 after irradiation). Animals were observed for 60 days with ANC measured every 1 to 2 days. The population model of ANC response to AR and the link between observed ANC time course and OS consisted of three submodels characterizing injury due to radiation, granulopoiesis, and a time-to-event model of OS.

**Results:**

The ANC response model accurately described the effects of AR exposure on the duration of neutropenia. ANC was a valid surrogate for survival because it explained 76% (95% CI, 41%–97%) and 73.2% (95% CI, 38.7%–99.9%) of the treatment effect for filgrastim and pegfilgrastim, respectively.

**Conclusion:**

The current model linking radiation injury to neutropenia and ANC time course to OS can be used as a basis for translating these effects to humans.

**Electronic supplementary material:**

The online version of this article (10.1007/s11095-020-02839-3) contains supplementary material, which is available to authorized users.

## Introduction

Acute radiation syndrome (ARS) is caused by exposure of large parts of the body to lethal amounts of penetrating radiation over a short period. Penetrating radiation can destroy bone marrow cells and lead to the hematopoietic syndrome of ARS (HS-ARS), a potentially fatal condition characterized by neutropenia, thrombocytopenia, and anemia (1). Granulocyte colony-stimulating factors (G-CSFs) such as filgrastim and pegfilgrastim have the potential to treat HS-ARS (2,3). G-CSF is a hematopoietic growth factor that stimulates the activation, proliferation, differentiation, and survival of mature neutrophils and neutrophil precursor cells (4). Filgrastim is a recombinant methionyl human G-CSF with a half-life of approximately 3.5 h (2,5). Pegfilgrastim is a long-acting G-CSF produced by covalently binding a 20-kD polyethylene glycol molecule to the N-terminal methionine residue of filgrastim with a half-life ranging from 15 to 80 h (3). Both filgrastim and pegfilgrastim have been found to decrease the depth and duration of neutrophil suppression during myelosuppressive chemotherapy and to reduce the incidence of infection characterized by febrile neutropenia (6–10). Therefore, administration of G-CSF therapy (filgrastim or pegfilgrastim) could potentially be useful in promoting recovery of absolute neutrophil count (ANC) as well as reducing the rates of infection and mortality in patients with HS-ARS.

Therapies for certain indications such as HS-ARS cannot be pursued through traditional human trials because they would be unethical. In such instances, well-controlled animal efficacy studies can act as a surrogate for an efficacy study in humans under the assumption that the benefit observed in these studies would likely translate into clinical benefits in humans (11). Two separate pivotal studies were conducted and showed that filgrastim and pegfilgrastim significantly decreased the duration of radiation-induced neutropenia and significantly improved overall survival (OS) in nonhuman primates (NHPs) that were exposed to acute radiation (12–14). Daily administration of 10 μg/kg filgrastim after acute radiation exposure significantly decreased the duration of grade 4 neutropenia (a reduction of 4.3 days in Farese *et al*., 2013 (14), and 8.9 days in Farese *et al*., 2012 (12)). Filgrastim also significantly improved 60-day OS by 38.3% compared with control subjects who received only supportive care (i.e., fluid support, antibiotics, analgesics, anti-diarrheals, antipyretics, anti-emetics, anti-ulceratives, nutritional support, and blood transfusions) (14). Similarly, weekly doses of 300 μg/kg pegfilgrastim for 2 weeks after acute radiation exposure shortened the duration of grade 4 neutropenia by 5 days (Hankey *et al*., 2015 (13)), and 14.1 days (Farese *et al*., 2012 (12)) and increased survival by 43.5% compared with controls (13).

To better translate the potential benefits of G-CSF to treat HS-ARS in humans, two aspects of this system must be quantified. First, it is necessary to model the myeloablative effects of lethal levels of irradiation on granulopoiesis in NHPs. This was achieved by adapting a structural model of chemotherapy-induced neutropenia (CIN) in humans (15). Second, a relationship between the ANC time course and OS must be identified. Both of these aspects are the objectives of the work presented herein.

## Materials and Methods

### ANC Response and Survival Data

Data on ANC responses in NHPs exposed to potentially lethal irradiation were taken from two pivotal studies evaluating the efficacy of filgrastim and pegfilgrastim on OS. Detailed methods have been published previously (13,14). Briefly, animals were randomly placed into either placebo or drug-treated groups and exposed to whole body irradiation (750 cGy at 80 cGy/min) on day 0. Filgrastim-treated animals were administered filgrastim 10 μg/kg daily (QD) subcutaneously (SC) starting on day 1; administration continued until ANC recovered to 1 × 10^9^ cells/L for 3 consecutive days. Pegfilgrastim-treated animals were administered pegfilgrastim 300 μg/kg SC on days 1 and 8 of the study. Samples for ANC quantification were collected pretreatment and every 1 to 2 days (duration = 60 days), and survival was evaluated longitudinally. Relevant information concerning the treatment cohorts is summarized in Table I. In the original animal studies (12–14) from which data were applied for the ANC time course modeling presented here, the research adhered to the “Principles of Laboratory Animal Care” (National Institutes of Health [US] publication #85–23, revised in 1985).

**Table I:** Summary Statistics of Subjects in the Filgrastim and Pegfilgrastim Pivotal Studies in NHPs Exposed to Lethal Levels of Radiation

Parameter	Filgrastim Pivotal Study			Pegfilgrastim Pivotal Study			Combined Studies		
	Placebo	Drug	Total	Placebo	Drug	Total	Placebo	Drug	Total
Subjects, n	22	24	46	23	23	46	45	47	92
ANC measurements, n	650	–	–	696	–	–	1346	–	–
Median body weight (SD), kg	6.2 (0.62)	5.8 (0.60)	5.9 (0.65)	6.2 (0.95)	5.8 (0.96)	6.1 (0.95)	6.2 (0.083)	5.8 (0.86)	5.9 (0.86)
Subjects receiving whole blood transfusions, n	21	21	42	23	23	46	44	44	88
Median baseline ANC (SD), 10^9^ cells/L	2.84 (1.54)	5.75 (1.58)	4.74 (2.15)	1.74 (1.23)	1.83 (1.89)	1.77 (1.59)	2.11 (1.46)	4.69 (2.47)	2.65 (2.21)
Male, n	18	20	38	23	23	46	41	43	84
Female, n	4	4	8	0	0	0	4	4	8

ANC, absolute neutrophil count; NHP, nonhuman primate; SD, standard deviation

### Structural Model

**Fig. 1:**
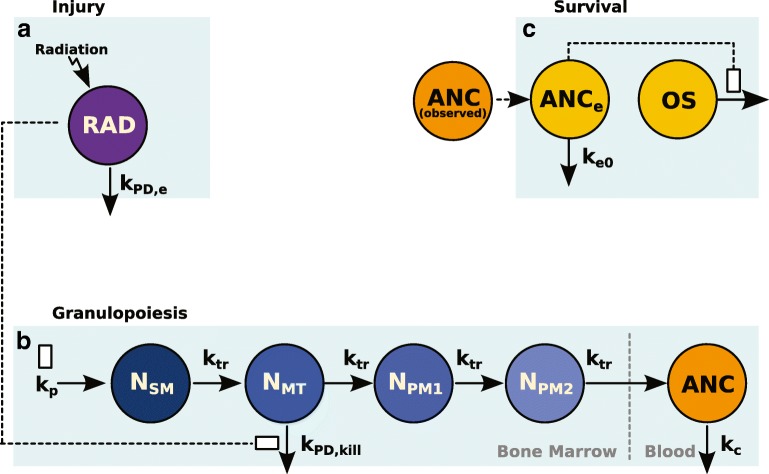
ANC response and OS model structure. Bone marrow stem cells (*N*_*SM*_) are produced nominally at a rate of *k*_*p*_. These cells mature through the mitotic (*N*_*MT*_) and precursor stages (*N*_*PM1*_ and *N*_*PM2*_) at a rate of *k*_*tr*_ and join neutrophils in the blood (*ANC*) with a turnover rate of *k*_*c*_. (**a**) Radiation exposure enters the *RAD* compartment, which diminishes at a rate of *k*_*PD,e*_ and (**b**) kills cells in the in the mitotic phase (*N*_*MT*_) at a rate of *k*_*PD,kill*_ proportional to *RAD* with sensitivity γ. Drug effect (not modeled here) is expected to stimulate both the production (k_p_) and maturation rates (k_tr_). (**c**) The observed ANCs drive an effect compartment (*ANC*_*e*_) that equilibrates at a rate of *k*_*e0*_*.* The ANC_e_ drives the survival model (OS). ANC, absolute neutrophil count; OS, overall survival; RAD, radiation.

A model of the effects of chemotherapy on granulopoiesis in humans was applied here to NHPs to model the effects of ARS (15). The structural model (Fig. 1) of ANC response to radiation was composed of three components. The first component accounts for the duration and magnitude of the radiation injury, which acts on the cells in the mitosis stage of granulopoiesis (Fig.1a). The second component was adapted from a model of CIN in humans and relates the radiation to ANC suppression (Fig. 1b). While the drug effects are not modeled explicitly, a decrease in the duration of neutropenia is assumed to result from increases in both the production rate of precursors and the maturation rate. The third component relates the ANC time course to OS (Fig. 1c). While the radiation and G-CSF in the drug-treated cohorts inputs are not represented explicitly when modeling survival, the OS model does contain this information implicitly as differences in the depth and duration of ANC suppression are expected to be driven by these two effects.

### Duration and Magnitude of Radiation Injury

The effect of radiation was implemented using a kinetic pharmacodynamics (K-PD) approach (16), which accounts for loss of cells due to the initial killing as well as the residual effects of radiation on granulopoiesis. The total radiation dose of 7.5 Gy (80 cGy/min) over approximately 9.4 min (13,14) was administered to the radiation component of the model (*RAD*), and the radiation effect decreased exponentially over time (*k*_*PD,e*_).

1 $$ \frac{dRAD}{dt}=-{k}_{PD,e} RAD $$

Similar to the human CIN model, radiation was assumed to affect cells in the mitotic phase (*N*_*MT*_), as shown in Eq. 2, where *k*_*kill*_, the rate of mitotic cell loss, is proportional (*k*_*PD,kill*_) to *RAD* with the sensitivity to radiation determined by γ.

2 $$ {k}_{kill}=-{k}_{PD, kill}{(RAD)}^{\gamma } $$

### Neutrophil Maturation, Response to Injury, and Treatment

The equations governing neutrophil dynamics are as given:

3 $$ \frac{d{N}_{SM}}{dt}={k}_p-{k}_{tr}{N}_{SM} $$

4 $$ \frac{d{N}_{MT}}{dt}={k}_{tr}{N}_{SM}-\left({k}_{tr}+{k}_{kill}\right){N}_{MT} $$

5 $$ \frac{d{N}_{PM1}}{dt}={k}_{tr}{N}_{MT}-{k}_{tr}{N}_{PM1} $$

6 $$ \frac{d{N}_{PM2}}{dt}={k}_{tr}{N}_{PM1}-{k}_{tr}{N}_{PM2} $$

7 $$ \frac{dANC}{dt}={k}_{tr}{N}_{PM2}-{k}_c ANC $$

The granulopoiesis model tracks cells in the bone marrow as they are produced as stems cells (*N*_*SM*_), enter mitosis (*N*_*MT*_), and mature through two precursor states (*N*_*PM1*_ and *N*_*PM2*_) before entering systemic circulation (*ANC*). The parameters *k*_*p*_ and *k*_*tr*_ define the proliferation rate of stem cells and the transit rate between compartments.

The rate of elimination of cells from the system is determined by *k*_*c*_. Initial values of the system at homeostasis are described as follows:

8 $$ {\displaystyle \begin{array}{c}{N}_{SM}(0)={N}_{MT}(0)={N}_{PM1}(0)={N}_{PM2}(0)=\frac{k_p}{k_{tr}}\\ {} ANC(0)=\frac{k_p}{k_c}\end{array}} $$

A time-to-event model with a time-varying hazard, *λ(t)*_*i*_, was used to characterize OS. The probability of the *i*-th subject to survive up to time t is given by *S(t)*_*i*_ as shown:

9 $$ S{(t)}_i={e}^{-{\int}_0^t\lambda {(t)}_i dt} $$

The time-dependent hazard function, *λ(t)*_*i*_, in the log domain was dependent on ANC as described by the following equation:

10 $$ \log \left(\lambda {(t)}_i\right)={\lambda}_{ANC}\frac{ANC_e{(t)_i}^{\lambda_{BC}}-1}{\lambda_{BC}} $$

where *λ*_*ANC*_ is a slope relating the hazard to a Box–Cox transformation of *ANC*_*e*_, and *λ*_*BC*_ is the power parameter of the Box–Cox transformation. *ANC*_*e*_ is the observed ANC delayed through an effect compartment as described by:

11 $$ \frac{dANC_{ei}}{dt}={k}_{e0}\  ANC{(t)}_i-{k}_{e0}{ANC}_{ei} $$

where *k*_*e0*_ is the equilibration rate for the effect compartment, and *ANC(t)*_*i*_ is the observed ANC in individual *i* at time *t*. At baseline, *ANC*_*ei*_ is set to the initial ANC value for individual *i*. At steady state, *ANC*_*e*_ equals the observed ANC.

### Statistical Model

Visual inspection of ANC time profiles suggested that the baseline ANC and rate of response to irradiation varied between subjects. As a consequence, interindividual variability (IIV) in model parameters for baseline ANC (*ANC*_*IC*_), precursor maturation rate (*k*_*tr*_), the duration of radiation effect (parameterized as an elimination rate*, k*_*PD,e*_), and the killing effect of radiation (*k*_*PD,kill*_) were included in the model and were assumed to be log-normally distributed in Eq. 12.

12 $$ {P}_i=P{e}^{\eta_i} $$

where *P*_*i*_ is the parameter for the *i*-th individual; *P* is the population typical value for the parameter; *η*_*i*_ is a random interindividual effect, and it is assumed to be a random normal variable with a mean of zero and variance ω^2^ that distinguished the *i*-th individual’s parameter from the population typical value, *P*, as estimated by the regression model. The magnitude of IIV in the system parameters was expressed approximately as a coefficient of variation (%CV).

Residual variability was evaluated using an exponential error model according to Eq. 13.

13 $$ \ln \left({Y}_{obs}\right)=\ln \left({Y}_{pred}\right)+\varepsilon $$

where *Y*_*obs*_ is the observed ANC concentration, and *Y*_*pred*_ is the corresponding model predicted ANC concentration. The residual error (*ε*) was assumed to be additive in the log domain and to follow a normal distribution with a mean of zero and variance σ^2^.

### Data Analysis

An exploratory analysis was performed to identify any trends present in the ANC time course, the response to radiation treatment, and any apparent study differences. Population analyses were performed using NONMEM® (version 7.2, ICON Development Solutions, Dublin, Ireland) on the high performance cloud computing system, Metworx™ v3.0 (Metrum Research Group, Tariffville, CT, USA). Metworx is a computational platform comprising a suite of software programming tools, procedures, and services that support population pharmacokinetic and pharmacodynamic data analyses. Graphical diagnostics and all other statistical analyses, including evaluation of NONMEM outputs, were performed with TIBCO Spotfire S + ® 8.2.0 (TIBCO Software Inc., Palo Alto, CA, USA) and R version 3.0.1. For the OS analysis, model diagnostics were run using PsN version 3.4.2 (17).

### Model Development

Placebo-treated NHP data from both the filgrastim and the pegfilgrastim pivotal studies (13,14) were used to estimate the model parameters described in Eqs. 3 through 7. During model development, the performance of the model was evaluated using standard goodness-of-fit metrics, including scatter plots of observed *versus* predicted individual ANC, conditional weighted residual *versus* the population prediction and time, and normalized prediction distribution errors *versus* the population prediction and time.

The model described in Eqs. 9 through 11 was used to characterize OS for the filgrastim and pegfilgrastim pivotal studies (13,14). The primary method for evaluating the model performance was evaluation of the visual predictive check (VPC) on Kaplan-Meier curves. Specifically, the predicted survival time course and OS (mean and CI) were compared with the observed data.

### Covariate Analysis

Covariates included demographic factors (baseline ANC, weight, and sex) and whole-blood transfusions. Categoric covariates were incorporated into the model as index variables. Continuous covariate effects (*θ*_*cov*_) on the population typical value (*P*) were included by normalizing the individual covariate (*cov*_*i*_) to a reference value (*cov*_*r*_). Assuming the covariate effect would be log-normally distributed, the following was used to calculate the typical value for an individual:

14 $$ {P}_i=P{e}^{\eta_j}{\left(\frac{{\mathit{\operatorname{cov}}}_i}{{\mathit{\operatorname{cov}}}_r}\right)}^{\theta_{cov}} $$

The improvement in model fit after incorporating the covariates was assessed by decreases in the objective function (>10.83, df = 1, *P* < 0.001), reduction in IIV and residual variability, reduction of standard errors, and examination of diagnostic plots as previously described.

### Model Evaluation

A nonparametric bootstrap analysis (18,19) was performed as an internal model evaluation technique (*n* = 1000 draws for ANC model and 5000 draws for OS model). If the parameter estimates fell into the 95% CI obtained from the bootstrap analysis, the model parameters were considered unbiased and identifiable.

For the ANC response model, VPCs were performed by simulation of 500 ANC concentration-time replicates using the parameters from the model, the original data set, and the associated covariates. The observed 5th, 50th, and 95th percentiles of the prediction-corrected ANC values were summarized, plotted over time, and compared graphically with simulated prediction-corrected ANC values.

For the OS model, VPCs were performed by simulation of 300 study replicates from the model, the original data set, and associated covariates. Full ANC profiles were not available in all NHPs because the ANC data were not obtainable after death. Therefore, the median observed ANC per treatment arm was used in the VPCs. The increase in survival in animals treated with G-CSF *versus* placebo is driven by the differences in median ANC profiles. The observed survival, represented using a Kaplan-Meier plot, was compared graphically with a 95% prediction interval based on the simulated replicates for the whole population and by treatment arm. To evaluate the appropriateness of ANC time course as a biomarker for survival, Li’s method for time-dependent covariates was used to evaluate the proportion of survival benefit attributable to treatment with filgrastim or pegfilgrastim (20).

## Results

### ANC Response and Survival Data

Relevant summary data describing the two pivotal studies of HS-ARS in NHPs are listed in Table I. The number of subjects in the placebo- and drug-treated cohorts was similar across studies; however, only the filgrastim pivotal study (14) had female subjects (*n* = 4 per cohort) (14). In both studies, body weight was similar in each cohort, and most subjects received whole-blood transfusions. ANC data from intensive sampling (every 1–2 days) were available in each study, with subjects in the filgrastim pivotal study having a higher median baseline ANC value (4.78 × 10^9^ cells/L; data on file) than those in the pegfilgrastim pivotal study (1.77 × 10^9^ cells/L; data on file).

**Fig. 2:**
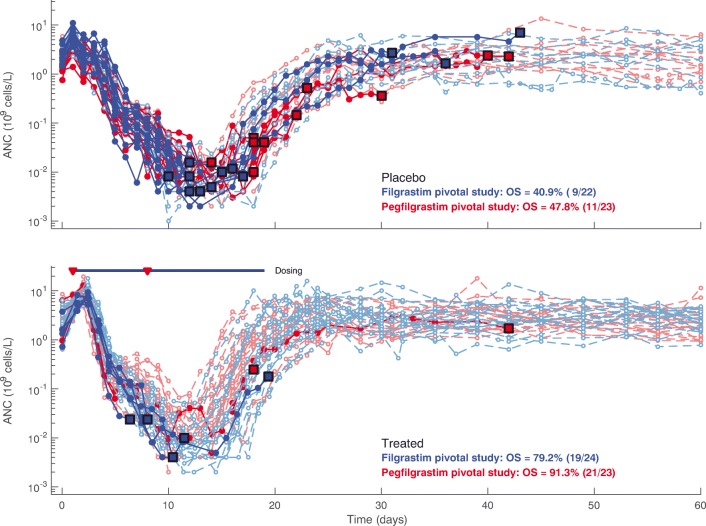
Exploratory data analysis of ANC response. ANC response in the filgrastim (blue) and pegfilgrastim (red) pivotal studies to whole body exposure of 750 cGy of radiation at 80 cGy/min on day 0 are shown. Each time series shows a single subject: dark lines and filled circular markers identify subjects that died during the study, and large square markers identify final ANC observation (time of death); light lines and open circular markers indicate animals that survived through day 60. The placebo cohorts are shown on the top panel, and the drug-treated cohorts (filgrastim or pegfilgrastim) are shown on the bottom panel. The median filgrastim dosing period (10 μg/kg QD from days 1–19, SC) is indicated by the horizontal solid blue line, and pegfilgrastim dosing (300 μg/kg on days 1 and 8, SC) is indicated by the red triangles. ANC, absolute neutrophil count; QD, daily; SC, subcutaneous.

Absolute neutrophil count time courses, stratified by treatment cohort and study, are shown in Fig. 2. Mortality rates in the placebo groups were similar between the filgrastim and pegfilgrastim studies. In both studies, mortality rate was lower in the groups treated with G-CSF compared with the respective placebo groups, and mortality rates were similar between the filgrastim and pegfilgrastim treatment arms. However, timing of deaths relative to the ANC nadir was markedly different between the two studies. In the filgrastim pivotal study (14), the 60-day overall mortality rate in the placebo group was 59.1% (13/22) (14), and six of the 13 animals that died on study were at their lowest observed ANC value (data on file). The 60-day overall mortality rate in the filgrastim-treated group was 20.8% (5/24) (14), and four of the five animals that died were at their individual ANC nadir (data on file). In the pegfilgrastim pivotal study (13), the 60-day overall mortality rate in the placebo group was 52.2% (12/23) (13), and 11 of the 12 animals that died did so after the ANC nadir (data on file). The 60-day overall mortality rate in the pegfilgrastim-treated group was 8.7% (2/23) (13), with the two animals dying several days after the ANC nadir had been reached (data on file).

### ANC Response

Together, the filgrastim and pegfilgrastim pivotal studies had 1346 measurements from the placebo cohort (Table I). During the estimation process, the ANC maturation rate (*k*_*tr*_) was found to correlate with baseline weight (BWT). Adding BWT as a covariate (Eq. 14) significantly improved the minimum value of the objective function (ΔMVOF = –36.7). None of the other covariates evaluated showed significant associations with any of the model parameters.

The estimated model parameters and their precisions are reported in Table II. Diagnostic plots (Fig. S1) displaying the observed *versus* population- and individual-predicted ANC showed random normal scatter around the identity line, indicating the absence of systematic bias and the adequacy of the model to describe the data at population and individual levels. The conditional weighted residuals also showed random normal scatter around zero with no specific pattern, suggesting no model misspecification. Moreover, the distribution of the conditional weighted residuals *versus* time remained relatively constant, indicating the absence of time-dependent bias in ANC predictions. The mean and standard deviation of the normalized prediction error (NPDE) were − 0.0800 (95% CI, −0.129 to −0.0314) and 0.893 (95% CI, 0.862 to 0.923), respectively. These results confirm the adequacy of the model to characterize the effects of acute radiation and filgrastim treatment on granulopoiesis in NHP, because the mean and standard deviation of the NPDE were close to the expected values of 0 and 1, respectively. Nevertheless, a slight trend to overpredict the variability was observed because the 95% CI of the NPDE standard deviation is <1. The histograms of the estimated random effects (Fig. S2) were centered and had acceptable shrinkage (<25% except for IIV on ANC_IC_). Correlations between random effects were relatively low (r^2^ < 0.16), except for *η*_*kPD,e*_ and *η*_*kPD,kill*_, which were already incorporated as covariance components in the model.

**Table II:** Parameter Estimates for the Final ANC Response Model

Parameter	Estimate	95% CI	RSE (%)
System Parameter			
*ANC*_*IC*_, 10^9^ cells/L	1.71	1.32, 2.10	11.6
*k*_*tr*_, d^−1^	0.628	0.608, 0.648	1.64
*k*_*c*_, d^−1^	1.34	0.892, 1.79	16.9
*k*_*PD,e*_, d^−1^	0.312	0.296, 0.329	2.69
*k*_*PD,kill*_, d^−1^KPD^−1^	2.14	1.43, 2.86	19.2
γ	1.79	1.61, 1.98	5.14
γ_wt_	0.629	–	Fixed
Between–Subject Variability			
*ω*_*ANCIC*_, %CV	23.1	17.0, 28.0	23.8
*ω*_*kPD,e*_, %CV	12	10.0, 13.7	15.3
*ω*_*kPD,kill*_, %CV	15.5	14.0, 17.0	9.8
*ω*_*kc*_, %CV	36.8	28.0, 43.8	21.8
Residual Error			
*σ*_*PE,*_ %CV	62	59.1, 64.8	4.64

*ANC*_*IC*_, baseline ANC; CV, coefficient of variation; *k*_*c*_, ANC turnover rate; *k*_*PD,e*_, elimination rate of radiation effect; *k*_*PD,kill*_, rate of cell loss due to radiation effect; *k*_*tr*_, precursor maturation rate; RSE, relative standard error; *γ*, radiation sensitivity; *γ*_*wt*_, sensitivity of *k*_*tr*_ on body weight; *σ*_*PE*_, proportional error; *ω*, variance and covariance for the subscripted parameters

The estimates and their corresponding precisions (RSE [%]) are those found from the population analysis. The remaining values (mean, CI) are the result of the nonparametric bootstrap

**Fig. 3:**
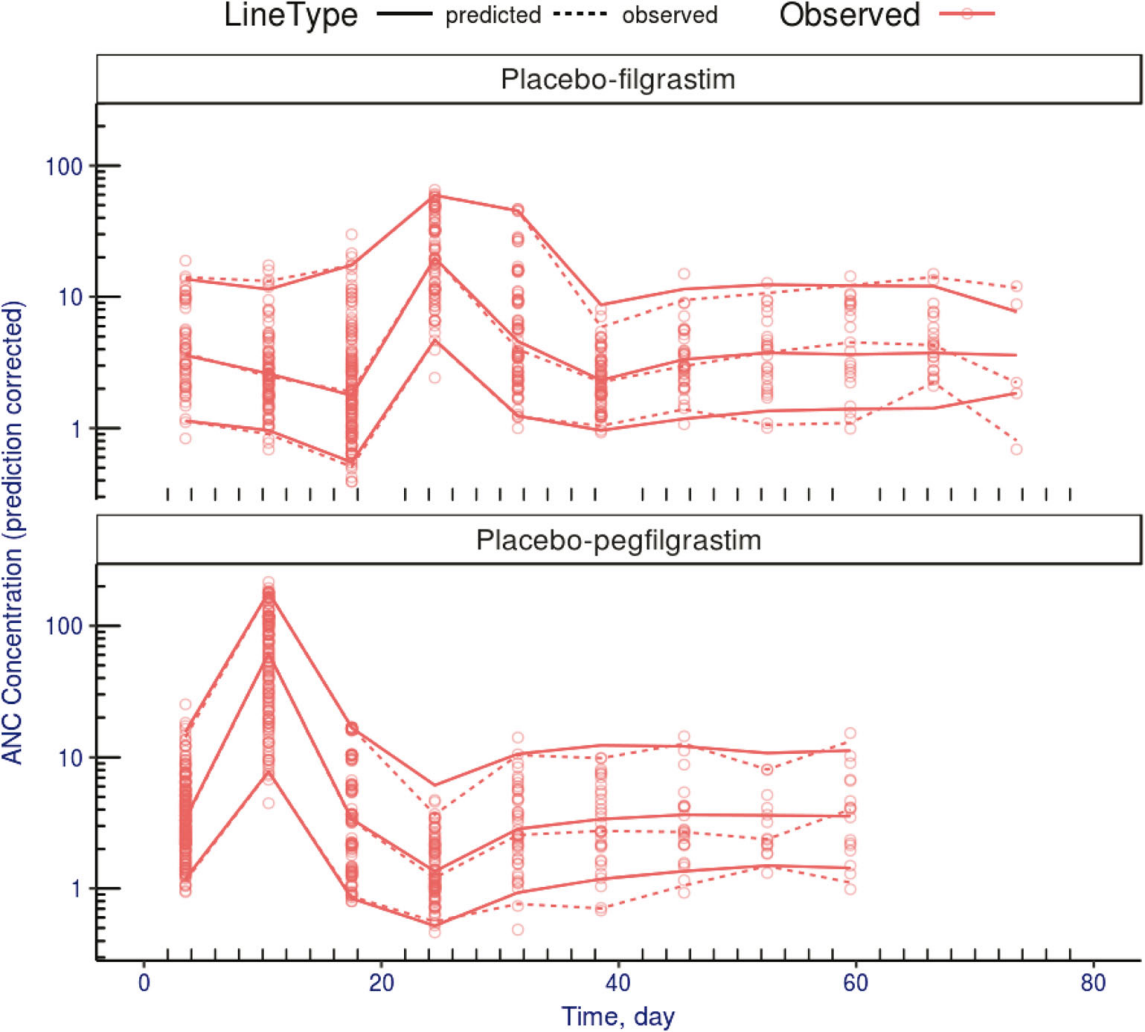
Visual predictive checks for the ANC response model: The placebo cohorts from the filgrastim (top) and pegfilgrastim (bottom) pivotal studies are shown. Lines show the model predictions (solid) and observations (dashed) for the 5th, 50th, and 95th data percentiles; and the markers show the binned data. The observed data are displayed as open circles. ANC, absolute neutrophil count.

The VPC of the placebo-treated cohorts is shown in Fig. 3. Parameter estimates and the summary statistics from the nonparametric bootstrap (467 replicates minimized successfully) are provided in Table II. Among all bootstrap replicates, the fixed and random effect parameters were very similar (<6% difference in the mean estimates). The fixed and random effect parameters were estimated with high to moderate precision (relative standard error [RSE] <25% for fixed effects and < 55% for random effects).

### Overall Survival

**Fig. 4:**
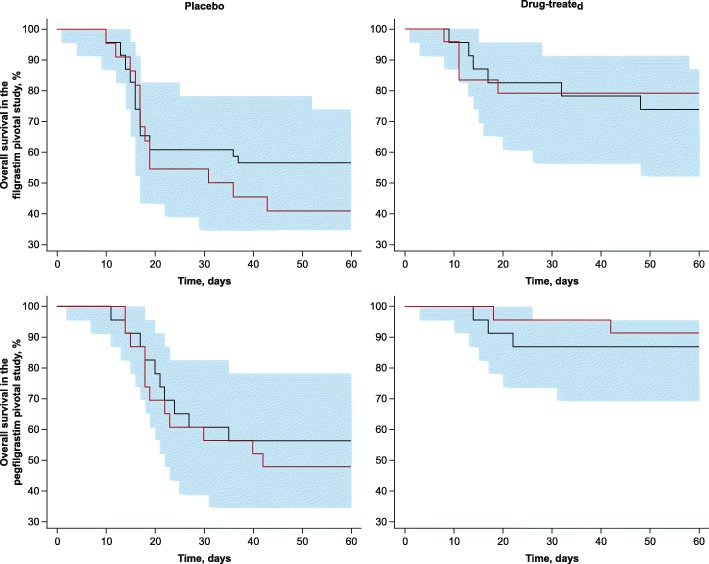
Visual predictive checks for OS model fitted separately to data from filgrastim (top panels) and pegfilgrastim (bottom panels) pivotal studies. The solid red line represents the observed survival for each cohort, and the black line provides the median model prediction. The shaded region represents the 95% prediction interval for the model. OS, overall survival.

The model described in Eqs. 9 through 11 used the observed ANC time courses as drivers of survival, delayed through an effect compartment. The extent of this delay was governed by the relative timing of the lowest ANCs and the times when the animals died. As identified in the exploratory analysis, whereas the time of the nadir was similar between studies (Fig. 2), the timing of deaths in both the placebo- and drug-treated cohorts relative to the nadir differed between studies (Fig. 4). Efforts to assign these differences to the available covariates were unsuccessful. An unexplainable study effect was found to exist, and study-specific parameter estimates were obtained to describe OS (Table III).

**Table III:** Parameter Estimates for the OS Model (Describing Survival as a Function of ANC)

	Filgrastim Pivotal Study			Pegfilgrastim Pivotal Study		
Parameter	Estimate	RSE (%)	95% CI	Estimate	RSE (%)	95% CI
*λ*_*ANC*_	−2.15	28	−3.32, −0.966	−0.229	58	−1.12, −0.00638
*λ*_*BC*_	−0.347	40	−0.616, −0.0772	0.3	39	−0.107, 0.915
*k*_*e0*_ (1/d)	0.668	6	0.592, 0.745	0.156	46	0.0221, 0.374

*λ*_*ANC*_, ANC effect on hazard (slope); *λ*_*BC*_, Box–Cox normalization factor; *k*_*e0*_, effect compartment equilibration rate; RSE, relative standard error

The estimates and their corresponding precisions (RSE [%]) are those found from the population analysis. CIs (95% CI) are the result of the nonparametric bootstrap

VPCs for both cohorts treated with placebo and G-CSF are shown in Fig. 4. VPCs for the OS model were fitted separately to data from the filgrastim pivotal study (top panels) and the pegfilgrastim pivotal study (bottom panels). ANC time course was found to explain 76% (95% CI, 41%–97%) and 73.2% (95% CI, 38.7%–99.9%) of the treatment effect for filgrastim and pegfilgrastim, respectively.

## Discussion

The objective of this work is to provide a bridge allowing the prediction of survival benefits of G-CSF therapy in human exposed to myeloablative radiation. Human models of granulopoiesis with G-CSF treatment in the CIN setting have been previously described (15,21). Two components are lacking in these previous models; the first is characterization of the radiation injury on cells in the bone marrow and the second is a link between ANC time course and OS. To this end, we developed a nonlinear mixed-effects model to quantify the effects of irradiation on ANC suppression and the link between ANC recovery and OS in a preclinical model of HS-ARS. The resulting model of granulopoiesis adapted an existing model of CIN to account for the radiation injury by quantifying the depth and duration of neutropenia (15). The VPC for the ANC response model (Fig. 3) shows good agreement with the fitted model predictions and the observed data.

The model predicts that the effects of radiation in the typical animal would decrease at a rate of *k*_*PD,e*_ = 0.312/d. This would translate into an effective half-life of approximately 2.2 days, and the underlying effects of radiation would persist for approximately 11 days (range, 10–12.5), assuming the washout period is equivalent to five effective half-lives. However, these effects would not be immediately reflected in the ANC because the radiation effect was assumed to be exerted on the neutrophil precursors in the mitotic stage, which are upstream in the chain of neutrophil production and release. In this context, the maturation rate (*k*_*tr*_ = 0.628/d) will determine the approximately 5.5-day lag between radiation exposure and systemic observations. Consequently, it is expected that ANCs will start decreasing 4 days after irradiation and will continue to decline for the next 11 days. Therefore, the ANC nadir is expected to be approximately 15 days after the radiation, which is consistent with the observable nadir at approximately 15 days (Fig. 2). The fitted value of γ = 1.79 indicates that the radiation effect is more than dose proportional. The injury from radiation can be related to chemotherapy by considering the duration and severity of the two scenarios. The effect of chemotherapy using the same granulopoiesis model was dose proportional and had a half-life of 3.5 h. The comparative predicted killing effect of radiation on mitotic cells is both immediate and more severe. The estimated proportion of cells killed by day 10 (postirradiation) was 99.33% compared with preradiation baseline as expected from an acute lethal radiation dose. Based on the current results, the estimated half-life in NHPs is 12 h (ln (2)/*k*_*c*_). Using their state-of-the-art technique, He *et al*. (22) reported an estimated half-life of blood-circulating neutrophils in rhesus macaques of approximately 1.63 days compared with previous estimates ranging between 7 h and 3.8 days in humans (23,24). The former study used radionuclide-labeled cells and measured clearance from the blood (23). He *et al*. noted that earlier studies on human neutrophil kinetics used adoptive transfer techniques or toxic radioactive labeling methods that likely altered the active state of neutrophils, resulting in an underestimated half-life of human neutrophils (22). *In vivo* studies that used nontoxic labeling methods estimated the half-lives of circulating neutrophils in humans at 13–19 h and the above mentioned 3.8 days (24,25). These more recent findings support that a human neutrophil half-life of 7 h was likely an underestimate and that our comparative NHP estimates based on our current data are acceptable. Such consideration is especially important when exploring the implications on the rate of neutrophil suppression and the onset of neutropenia in humans compared with NHPs.

A number of relationships (linear, log-linear, logit, and box-cox) were considered to characterize the survival of NHPs in response to radiation (both with and without treatment with G-CSF). Using predicted ANC values in the effect compartment as inputs into the time-varying hazard of the OS model was found to best characterize NHP survival after acute lethal radiation both with and without exogenous filgrastim or pegfilgrastim. The exploratory analysis identified important interstudy differences in the time course of OS. In the filgrastim pivotal study (14), most deaths occurred at or near the nadir, whereas in the pegfilgrastim pivotal study (13), most deaths occurred during the neutrophil recovery phase. The susceptibility of an animal to opportunistic infections increases with ANC suppression, and the time course of a given pathogen-induced disease may vary widely. Despite efforts to control experimental conditions, it is possible that the two groups of animals were exposed to different sets of environmental challenges leading to death at different times. An effect compartment was used to delay the two related signals; ANC ultimately contributes to the OS part of the model. The delay also accounts for the discrepancy between the time of the nadir and the actual death of animals. Consequently, the OS model described by Eqs. 9 through 11 was fitted separately to each data set, resulting in an equilibration half-life (estimated as ln (2)/*k*_*e0*_) of 1.0 day for the filgrastim pivotal study, but 4.4 days for the pegfilgrastim pivotal study. Because the model is not at steady state, the ANC concentrations in the effect compartment are different from those measured systematically. Consequently, the scaling factor (λ_ANC_) was different between studies (at −2.145 and − 0.229 for the filgrastim and pegfilgrastim pivotal studies, respectively). The range of values in these parameters represents the temporal differences in the observed mortality and reflects the varying impact of neutrophil suppression on time of death. However, OS at the end of the study is not impacted.

The time course of neutropenia was used as an input to a time-to-event model describing the time course of survival. As Fig. 4 shows, this model accurately captures the time course of survival for both placebo- and drug-treated animals. The observed OS data from NHPs exposed to lethal doses of radiation suggest that exogenous G-CSF supplementation provides a survival benefit. The relative risk reduction in mortality at 60 days postirradiation was found to be 64% for filgrastim and 82% for pegfilgrastim. The majority of the treatment effect on OS could be explained by the time course of ANCs, which suggests that ANC is an adequate marker to explain and predict the benefits of treatment with either filgrastim or pegfilgrastim. Accordingly, ANCs can be used as a translational surrogate for predicting the survival benefit of filgrastim or pegfilgrastim when scaling results from animals to humans.

## Conclusion

These results support utility of the model to quantify the effects of acute lethal radiation in NHPs. Similarly, the OS model establishes a quantifiable and predictive link between ANC time courses and NHP survival after exposure to lethal levels of radiation and accounts for the treatment effects of filgrastim or pegfilgrastim on OS. While this model does not take into account the target- mediated effects of G-CSF treatment on pharmacokinetics and the explicit effects on ANC response, there are clinical models available to inform these system-specific human parameters. Taken together, the model of ANC response and OS in NHPs can be combined with clinical models of filgrastim or pegfilgrastim used to treat hematopoietic injuries to predict the potential survival benefits of filgrastim or pegfilgrastim in humans with HS-ARS.

### Acknowledgments and Disclosures

This work was funded by Amgen Inc. The authors would like to thank Ann Farese from the University of Maryland for explaining the nuances of the pivotal NHP studies. We would also like to acknowledge the work of Yihan Jiang and Bo Zhang from Amgen Inc. for their work in creating NONMEM data sets for the analysis. Medical writing support was provided by Meghan Johnson, PhD, and James Balwit, MS, CMPP (Complete Healthcare Communications, LLC, North Wales, PA, USA), whose work was funded by Amgen Inc. DATA SHARING: Qualified researchers may request data from Amgen clinical studies. Complete details are available at the following: http://www.amgen.com/datasharing. Conflicts of Interest: J.H. was an employee of Amgen Inc. at the time of this work and owns Amgen stocks; the current affiliation for J.H. is Seattle Genetics, Bothell, WA, USA. P.O.G has received consulting fees from Amgen; the current affiliation for P.O.G is POG Pharmacometrics, Hampshire, United Kingdom. I.D. has received consulting fees from MnS SPRL. P.J. has received consulting fees from MnS SPRL. J.J.P.R. was an employee of Amgen Inc. at the time this work was conducted and owns Amgen stock; the current affiliation for J.J.P.R. is Janssen Research & Development, Valencia, Spain. A.N. is an employee of Amgen Inc. and owns Amgen stocks. S.D. is an employee of Amgen Inc. and owns Amgen stocks. A.C. was an employee of Amgen Inc. at the time this work was conducted, owns Amgen stock, and has Amgen stock options; the current affiliation for A.C. is Rigel Pharmaceuticals Inc., South San Francisco, CA, USA. B-B.Y. is an employee of Amgen Inc. and owns Amgen stocks. M.M. was an employee of Amgen Inc. at the time this work was conducted and owns Amgen stock; the current affiliation for M.M. is Vertex Pharmaceuticals, Boston, MA, USA. Ethical Approval: This article does not contain any studies with animals performed by any of the authors.

## Electronic supplementary material

ESM 1 (PDF 577 kb)

## Abbreviations

ANC: Absolute neutrophil count
ARS: Acute radiation syndrome
BWT: Baseline weight
CI: Confidence interval
CIN: Chemotherapy-induced neutropenia
%CV: Coefficient of variation
G-CSF: Granulocyte colony-stimulating factor
HS-ARS: Hematopoietic syndrome of acute radiation syndrome
IIV: Interindividual variability
K-PD: Kinetic pharmacodynamics
MVOF: Minimum value of the objective function
NHP: Nonhuman primate
NPDE: Normalized prediction error
OS: Overall survival
RSE: Relative standard error
SC: Subcutaneous
QD: Daily
VPC: Visual predictive check
