# Supplementary material for: Quantification of Radiation Injury on Neutropenia and the Link between Absolute Neutrophil Count Time Course and Overall Survival in Nonhuman Primates Treated with G-CSF
Source: Pharm Res. 2020 May 21;37(6):102. doi: 10.1007/s11095-020-02839-3 (PMC7242243; doi:10.1007/s11095-020-02839-3)
Supplement: Supplementary file 1 — (PDF 577 kb) [file 11095_2020_2839_MOESM1_ESM.pdf]

Quantification of Radiation Injury on Neutropenia and the Link Between Absolute Neutrophil Count Time Course and Overall Survival in Nonhuman Primates Treated With G-CSF

Pharmaceutical Research

John Harrold,[1] Per Olsson Gisleskog,[2*] Isabelle Delor,[2] Philippe Jacqmin,[2] Juan Jose Perez-Ruixo,[1†] Adimoolam Narayanan,[1] Sameer Doshi,[1] Andrew Chow,[1‡] Bing-Bing Yang,[1] Murad Melhem[1§]

[1]Department of Clinical Pharmacology, Modeling and Simulation, Amgen Inc., Thousand Oaks, CA, USA; [2]SGS Exprimo NV; Mechelen, Belgium
*Affiliation at time of study; current affiliation is POG Pharmacometrics, Hampshire, United Kingdom.
[†]Affiliation at time of study; current affiliation is Janssen Research & Development, Valencia, Spain.
[‡]Affiliation at time of study; current affiliation is Rigel Pharmaceuticals Inc., South San Francisco, CA, USA.
[§]Affiliation at time of study; current affiliation is Vertex Pharmaceuticals, Boston, MA, USA.

Corresponding Author: Murad Melhem, PhD
Clinical Pharmacology
Vertex Pharmaceuticals
50 Northern Ave
Boston, MA 02210
Tel: 1 (617) 961-8788
Email: murad_melhem@vrtx.com

Supplementary Materials

Fig. S1. Goodness-of-fit plots for the ANC response model. The predicted versus observed for population (top, left) and individual (top, right) predictions; conditional weighted residuals are shown as a function of the population prediction (middle, left) and of the observed time (middle right); and the NPDE is provided as a function of the population prediction (bottom, left) and of the observed time (bottom, right). ANC, absolute neutrophil count; NPDE, normalized prediction error

Fig. S2. Distribution of interindividual variability parameters for ANC_{IC}, $k_{PD,e}$, $k_{PD,kill}$, and k_{tr}, with the shrinkage for each distribution shown in parentheses. The solid blue lines illustrate the density of the random effect distributions, the red vertical lines show medians of these distributions, and the dashed red lines show the density of the random effect distributions as estimated by the model. ANC, absolute neutrophil count; ANC_{IC}, baseline ANC; $k_{PD,e}$, duration of radiation effect; $k_{PD,kill}$, killing effect of radiation; k_{tr}, precursor maturation rate

Fig. S1

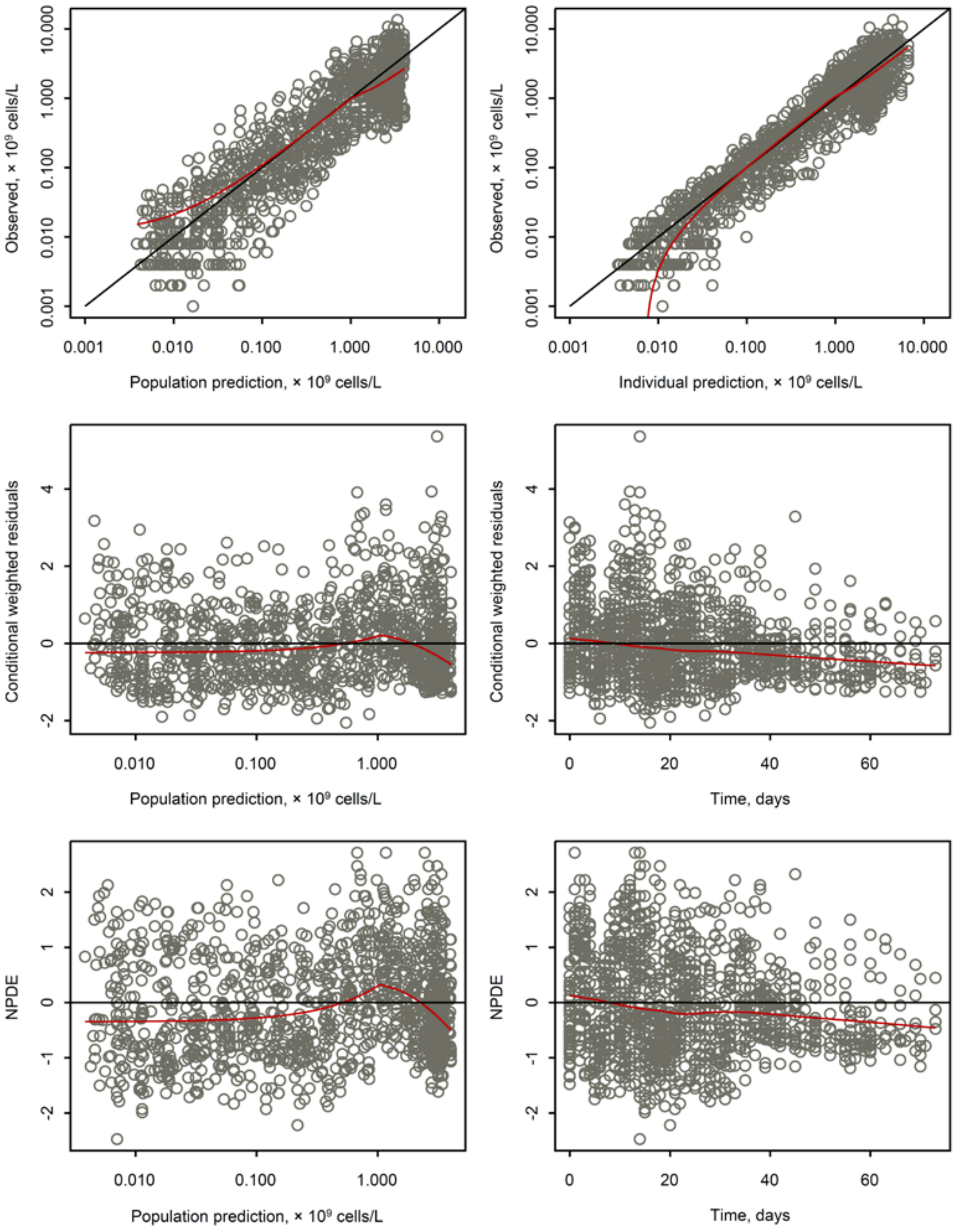
Observed, × 10⁹ cells/L
Population prediction, × 10⁹ cells/L
Individual prediction, × 10⁹ cells/L
0.001
0.010
0.100
1.000
10.000
Conditional weighted residuals
Time, days
NPDE
0
20
40
60
-2
-1
1
2
4

Fig. S2

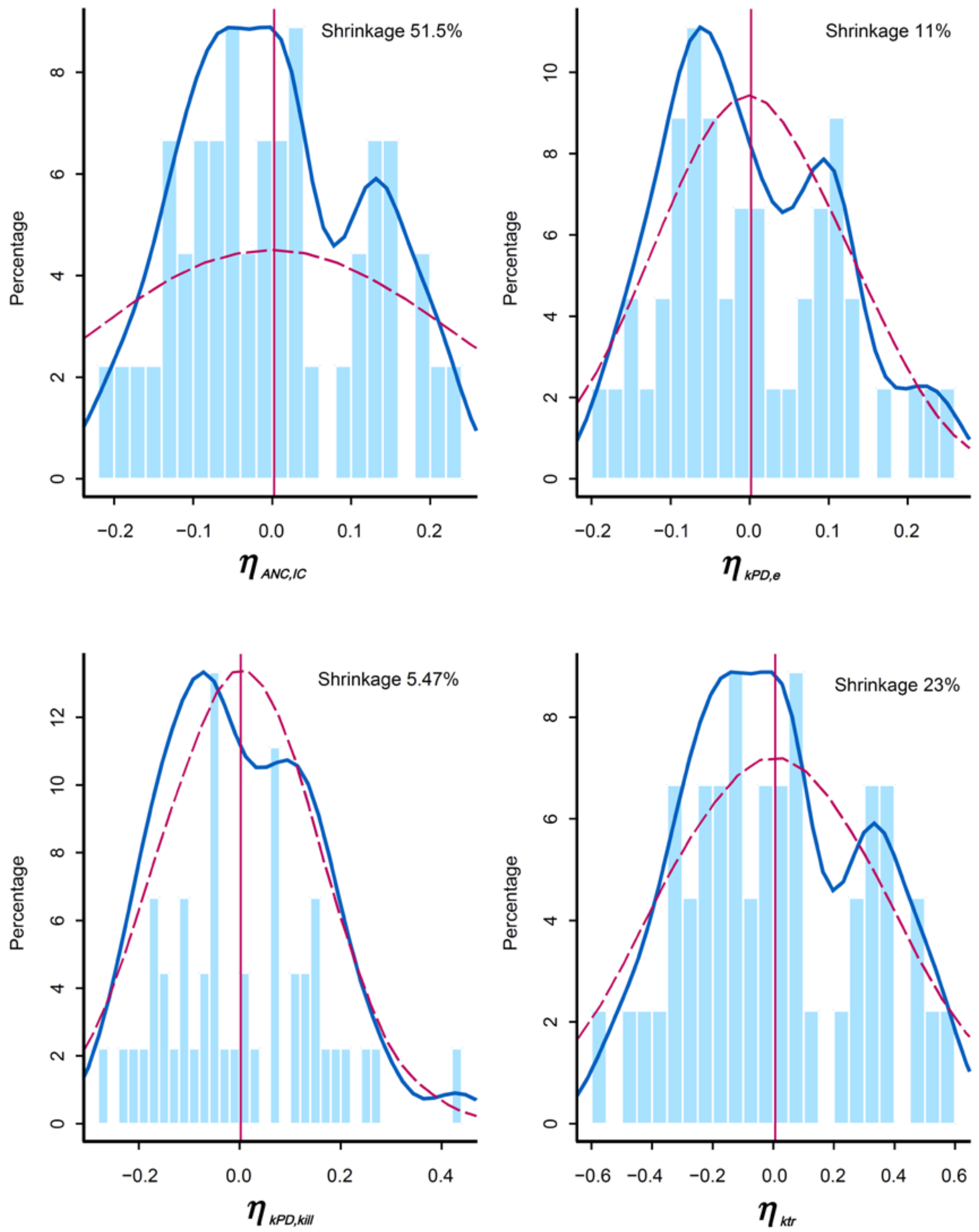
Shrinkage 51.5%
Percentage
$\eta_{ANC,IC}$
Shrinkage 11%
Percentage
$\eta_{kPD,e}$
Shrinkage 5.47%
Percentage
$\eta_{kPD,kill}$
Shrinkage 23%
Percentage
η_{ktr}
